# Draft Genome Sequence of a Novel Culturable Marine Chroococcalean Cyanobacterium from the South Atlantic Ocean

**DOI:** 10.1128/genomeA.00384-15

**Published:** 2015-04-23

**Authors:** Janaina Rigonato, Danillo O. Alvarenga, Luis H. Z. Branco, Alessandro M. Varani, Frederico P. Brandini, Marli F. Fiore

**Affiliations:** a University of São Paulo, Center for Nuclear Energy in Agriculture, Piracicaba, São Paulo, Brazil; b São Paulo State University, Institute of Bioscience, Languages and Exact Sciences, São José do Rio Preto, São Paulo, Brazil; c São Paulo State, University, Faculdade de Ciências Agrárias e Veterinárias, Jaboticabal, São Paulo, Brazil; d University of São Paulo, Oceanography Institute, São Paulo, Brazil

## Abstract

The novel chroococcalean cyanobacterium strain CENA595 was isolated from the deep chlorophyll maximum layer of the continental shelf of the South Atlantic Ocean. Here, we report the draft genome sequence for this strain, consisting of 60 contigs containing a total of 5,265,703 bp and 3,276 putative protein-coding genes.

## GENOME ANNOUNCEMENT

Coccoid cyanobacteria are important primary producers in the oceans, and the most known genera are *Synechococcus* and *Prochlorococcus* (1) from the order *Synechococcales*. Nevertheless, metagenomic studies revealed that several marine taxa remain undescribed (2), mainly due to the notorious difficulty in obtaining culturable strains. These unknown cyanobacteria, regardless of their abundance, may also have important roles in marine ecosystems. In this work, a novel coccoid cyanobacterial strain of the order *Chroococcales* was isolated from a water sample collected at the deep chlorophyll maximum layer (107-m depth) in the continental shelf of the Southwest Atlantic Ocean (25°15.595′S 045°07.670′W). The sampling cruise was carried out aboard the R/V Alpha Crucis of the University of São Paulo in November 2012. The water sampled was filtered through a 0.22-µm membrane, which was inoculated in ASM-I culture medium (3) prepared with 75% filtrated seawater. After a unicyanobacterial culture was obtained, genomic DNA was extracted using the PowerSoil DNA extraction kit (MoBio Laboratories, USA) and quantified using the Qubit Fluorometer (Life Technology, USA). Whole-genome sequencing was performed with the MiSeq platform (Illumina, USA) using the 600-cycle MiSeq reagent kit v3 (Illumina). Initially, the obtained paired reads were merged with PEAR 0.9.5 (4). Bases with quality scores under Phred 30 and sequences shorter than 50 bp were removed with seqyclean 1.9.8 (https://bitbucket.org/izhbannikov/seqyclean). *De novo* genome assembly was carried out with SPAdes 3.1.1 (5). Assembly statistics were obtained with QUAST 2.3 (6). The final draft genome assembly consisted of 127-fold average coverage and 60 contigs (>500-bp length), with a total size of 5,265,703 bp, an *N*_50_ value of 156,240, and a mean GC content of 42.60%. The automatic annotation using Prokka 1.10 (7) predicted 4,932 coding sequences, 60 tRNA genes, and 1 rRNA gene. antiSMASH 2.0 (8) detected 11 secondary metabolite gene clusters. The RAST annotation system (9) predicted 388 subsystems, which represent only 32% of the assigned sequences. Genes encoding osmoregulation, oxidative stress, heat shock, persister cells, UV-absorbing secondary metabolites, auxin biosynthesis, and bacterial chemotaxis, among others, were found. BLAST analysis of the 16S rRNA gene sequence revealed low identity (≤94%) with already known cyanobacteria. The genome sequence of this cyanobacterium brings new insights into the current classification of the coccoid group and in the reconstruction of the coevolutionary history of ecologically linked marine cyanobacteria based on their phylogenetic information.

### Nucleotide sequence accession numbers. 

The strain CENA595 genome sequence has been deposited in DDBJ/EMBL/GenBank under the accession number JYON00000000. The version described in this paper is version JYON01000000.
